# Cholinergic Urticaria: Clinical Presentation and Natural History in a Tropical Country

**DOI:** 10.1155/2020/7301652

**Published:** 2020-05-24

**Authors:** Chuda Rujitharanawong, Papapit Tuchinda, Leena Chularojanamontri, Nattacha Chanchaemsri, Kanokvalai Kulthanan

**Affiliations:** Department of Dermatology, Faculty of Medicine Siriraj Hospital, Mahidol University, Bangkok, Thailand

## Abstract

**Background:**

Cholinergic urticaria (CholU) is a subset of chronic inducible urticaria characterized by the recurrent pinpoint-sized wheals that are induced by exercising or increasing core body temperature. Currently, the data of CholU in tropical climate is still limited.

**Objective:**

To investigate the clinical features and natural course of CholU in a tropical country.

**Materials and Methods:**

This retrospective chart review study analyzed the data of CholU patients aged over 18 years who visited Siriraj Urticaria Clinic, Siriraj Hospital, Bangkok, Thailand, between January 2007 and September 2019. Demographic data, clinical presentations, and results of provocation tests and other laboratory investigations were evaluated and compared with other studies reported in temperate zones.

**Results:**

Sixteen out of 2,175 chronic urticaria patients (0.7%) were diagnosed with CholU. The median age of CholU patients was 28.0 ± 11.7 years with male predominance (56.3%). Three patients (18.8%) had a history of atopy. Fifteen patients (93.8%) were positive to the exercise provocation test. Nonsedating antihistamine drugs were a main treatment (73.8%). Six patients (37.5%) were in remission at the time of the study, with a mean duration 4.3 years. The Kaplan-Meier survival analysis demonstrated that 12.5%, 35.5%, and 67.9% of patients would have disease remission within 1 year, 5 years, and 13 years, respectively.

**Conclusions:**

The prevalence of CholU differs in each geographic region and is found to be low in tropical countries with a median duration 4.3 years. The prevalence of atopy and anaphylaxis with CholU is also lower in tropical countries than in temperate.

## 1. Introduction

Cholinergic urticaria (CholU) is a variant of inducible urticaria induced by a rising core body temperature after exercising, sweating, eating of spicy foods, stress, or passive warming [1]. CholU mostly appears in pinpoint-sized and urticaria eruptions with severe itch localized to the trunk and limbs and usually disappears rapidly on its own within a few hours. However, most patients with CholU feel discomfort in the skin that disturbs their quality of life [2–4]. In severe patients, CholU may be related to exercise-induced anaphylaxis, a severe wheal-flare reaction with systemic involvement including difficult breathing, wheezing, or abdominal pain [5]. The reported prevalence of CholU in a general population varies from 0.023% to 11.2% in temperate zone countries [6–8]. CholU mostly affects patients with age onset in their second to third decade [9]. The pathogenesis of CholU is still unclear. Some CholU patients are occasionally associated with anhidrosis/hypohidrosis [10]. Acetylcholine is noted to induce both sweating and wheals after intradermal injection [11]. The sweating-associated urticaria has been proposed as the etiology of CholU. In addition, sweat allergy that is a type I hypersensitivity against a component of sweat is observed in patients with CholU [12].

The diagnosis of CholU is established by history and an appropriate provocation test that is suitable to the patient's age and general condition. Previously, both heavy exercise and the passive warming that can increase the core body temperature have been utilized to confirm the diagnosis of CholU. CholU is diagnosed if wheal-flare reaction develops instantly or within a few minutes after heavy exercise or passive warming and commonly disappears within 15-60 minutes [13]. The intradermal injection of 100 *μ*g of methacholine in 0.1 ml of saline solution was used as another test for CholU diagnosis in both clinical and research settings. Wheal-flare reaction that occurs within 1 minute of the injection indicates a positive reaction [14, 15]. However, the positive methacholine skin test demonstrates a hypersensitivity to cholinergic mediators that is not specific to CholU [16]. Thus, it has become less popular. Nowadays, the European Academy of Allergology and Clinical Immunology Dermatology Section, the Global Allergy and Asthma European Network, the European Dermatology Forum, and the Urticaria Network e.V. (EAACI/GA^2^LEN/EDF/UNEV) recommends 2 methods to investigate patients who are suspected CholU: (i) exercise machines: patients exercise by a bicycle trainer or treadmill for 30 minutes resulting in an increased pulse rate by 3 beats/minute every minute, and while a patient is testing, the doctor will monitor signs of CholU; (ii) a passive warming test: a person stays in a 42 degrees Celsius (°C) bath, and a doctor monitors body temperature [1]. Patients continue the passive warming test for 15 minutes after body temperature increases ≥1°C over baseline. The positive result shows pinpoint-sized wheals during the test and ten minutes after ending the test [1].

The current EAACI/GA^2^LEN/EDF/UNEV consensus recommended the use of nonsedating histamine H_1_ receptor antagonist (nsAH_1_) drugs as the first-line treatment, together with trigger avoidance as the best approach for CholU [1]. In refractory cases, updosing of nsAH_1_ and omalizumab (a recombinant humanized IgG_1_ monoclonal antibody that binds to IgE) is recommended to improve the disease activity [1]. The addition of a H_2_ receptor antagonist (AH_2_), propranolol (*β*_2_-adrenergic blocker), montelukast, and botulinum toxin injection has been reported as an effective treatment [1]. Furthermore, desensitization by routine exercise or treatment with autologous sweat in CholU patients with sweat allergy has been reported. Nevertheless, desensitization procedure should be conducted under physician observation due to risk of anaphylaxis [12, 17, 18]. Relating to the disease courses, the reported mean disease duration in patients who had disease remission was 4-7.5 years [1, 9, 19].

At present, there were only 2 studies of CholU in tropical countries. Godse et al. demonstrated the prevalence of CholU in Indian students by conducting a survey questionnaire [6]. Sánchez et al. reported physical and environmental factors which played a key role in CholU by questioning Colombian patients about triggers with exacerbation of CholU [19]. However, the mean duration of disease remission and remission rate had never been reported. Thus, the information of CholU in tropical climate is still limited. This study is aimed at investigating the clinical features and natural history of CholU in Thailand which is a tropical country.

## 2. Materials and Methods

This retrospective chart review study was designed to analyze preexisting data of CholU patients aged over 18 years who visited Siriraj Urticaria Clinic, Siriraj Hospital, Bangkok, Thailand, between January 2007 and September 2019. The study received the permission from the Siriraj Institutional Review Board. Demographic data, clinical presentations, and results of provocation tests and other laboratory investigations were analyzed.

In this study, CholU was diagnosed by history of wheal-flare reaction and/or angioedema after exercise or increasing of the core body temperature and confirmed by provocation testing that matched the patient's age and general condition. The stationary bicycle was our routine provocation device. If patients were limited of physical activity, a passive warming test was performed by full bath at 42°C for up to 15 minutes until body core temperature increased more than 1°C [1]. The positive result was the appearance of present tiny wheals that often localized on the limbs and trunk within 10 minutes [1]. All patients should discontinue any antihistamines at least for 1 week before the confirmation tests.

A remission of CholU was defined as an absence of wheal-flare reaction or angioedema regardless of increasing the body temperature and discontinuation of any urticaria treatment for at least 6 months. Of the remission cases, the disease duration was determined from the onset of symptoms to the remission date.

### 2.1. Statistical Analysis

In descriptive statistics, the demographic data, disease duration, and results of provocation testing and laboratory investigations were illustrated by mean, minimum, maximum, standard deviation, frequency, and percentages. The probability of remission among patients with CholU at each time point was determined by a Kaplan-Meier survival curve. The analysis of statistical data was assessed by the Statistical Package for the Social Sciences (SPSS) Statistics for Windows, version 18 (SPSS Inc., Chicago, IL, USA).

## 3. Results

Out of the 2,175 chronic urticaria patients who visited Siriraj Urticaria Clinic between January 2007 and September 2019, 178 patients (8.2%) were diagnosed as inducible urticaria. Sixteen patients (0.7%) with CholU diagnosis were enrolled in this study. The mean age was 28.0 ± 11.7 years (range 18-56 years). The majority were males (56.3%).  Table 1 shows the clinical characteristics of our CholU patients. Concomitant CSU was detected in 4 patients (25.0%). One patient (6.3%) has CholU coexisting with angioedema. No patients had symptoms of anaphylaxis. Three patients had positive atopic history (18.8%), mostly allergic rhinitis (*n* = 2, 12.5%). All patients were healthy with no known underlying disease.

All patients were investigated by the exercise provocation test. Fifteen patients (93.8%) showed positive wheal and flare reaction. The only one patient with a negative exercise provocation test result had a confirmed history of wheal and angioedema after heavy exercise such as running and dancing.

**Figure 1:**
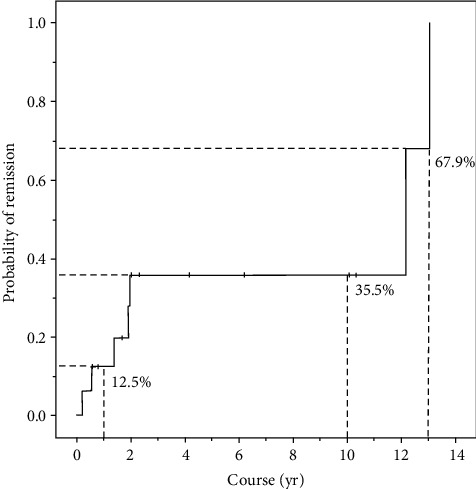
A Kaplan-Meier survival analysis (one minus survival function) demonstrated the remission during the first 2 years of CholU that was revealed in 5 (31.3%) patients. In addition, 2 (12.5%), 5 (35.5%), and 6 (67.9%) of patients were in remission after 1, 5, and 13 years, respectively, from the onset of CholU symptoms. The median duration when half of patients of CholU were in remission was 12.2 years. Abbreviations: CholU: cholinergic urticaria; yr: year.

Most of our CholU patients (73.8%) were treated with nonsedating second-generation H_1_ antihistamine drugs (nsAH_1_), i.e., fexofenadine (12 patients, 75%), cetirizine (7 patients, 43.8%), loratadine (6 patients, 37.5%), desloratadine (2 patients, 12.5%), bilastine (2 patients, 12.5%), and levocetirizine (1 patient, 6.3%). (Table 1) The dosage was ranged from standard up to 2-fold. Before the year 2014 when the EAACI/GA^2^LEN/EDF/UNEV consensus panel recommendations had been established, 4 patients had taken one or combination of nsAH_1_. Of sedating antihistamine drugs, hydroxyzine was the most common prescribed drug (4 patients, 25%). Four patients (25%) were treated with the combination of nsAH_1_ and sAH_1_. The recalcitrant patients to AH_1_ drugs needed other medications to control symptoms including ranitidine (4 patients, 25%), cimetidine (1 patient, 6.25%), propranolol (1 patient, 25%), chloroquine (1 patient, 25%), and colchicine (1 patient, 25%). Six patients (37.5%) were in remission at the time of the study, with a mean disease duration 4.3 ± 4.5 years (range 0.3-13.0 years) as shown in Table 1. As stated in the Kaplan-Meier survival analysis, 5 patients (31.3%) were in remission during the first 2 years of the disease.  Figure 1 demonstrates that the remission within 1 year, 5 years, and 13 years was 12.5%, 35.5%, and 67.9% of patients, respectively. After onset, the median duration before half of the patients were in remission was 12.2 years.

## 4. Discussion

In this study, the prevalence of CholU among chronic urticaria patients in a tropical country is 0.7%. Our group previously reported the prevalence of CholU among chronic urticaria patients of 0.9% and 0.5% in the years 2007 and 2011, respectively [20, 21]. The reported prevalence of CholU among general population in countries located in the tropical zones ranged from 0.023 to 11.2% [6–8]. Most studies including our study have shown the male predominance [6]. The mean age at symptom onset in our study was 28.0 ± 11.7 years that is consistent with the data reported in temperate zones [9]. Ten patients (62.5%) had concomitantly other types of urticaria, mostly CSU. One patient had a combination of CholU, CSU, and dermographism.

Table 2 presents the information of CholU obtained in 3 temperate countries and 2 tropical countries compared with our study [2, 6–9, 19]. Although Thailand is located in the tropical zone and the latitude here seems to induce CholU, the prevalence of CholU is lower than in other temperate countries. We proposed that patients in the tropical zone are used to be in hot environment and the severity of CholU may be mild. Some patients misunderstand that the wheal-flare reaction is caused by sweating or irritation due to dust and do not see the doctor. Thus, the prevalence of CholU may be underdiagnosed in the tropical zone. Duke proposed that changes in temperature might be an associated factor in temperature-related urticaria, i.e., cold urticaria, rather than constant temperature [22]. Perhaps, the prevalence of CholU is less frequent in tropical countries due to less change in hot temperature. However, the supporting knowledge is still deficit to support that changes in temperature are likely to induce temperature-related urticaria (cold urticaria, heat urticaria, and CholU).

In this study, 3 patients (18.8%) had positive histories of atopy, i.e., allergic rhinitis (2 patients, 12.5%) and asthma (1 patient, 6.3%). In 2012, Vichyanond et al. reported the prevalence of allergic rhinitis and asthma in healthy subjects (26.3% and 8.8%, respectively) [23]. Nowadays, the prevalence of atopy trends to increase throughout the world [24]. These data implied that the prevalence of atopy was not raised in CholU patients in our study. Contrary to the studies of temperate countries, Zuberbier et al. reported the high prevalence of atopy in German patients with CholU (45.5%) to be statistically significant demonstrated when compared with control persons (30.8%) (*p* < 0.05) [7].

None of patients in our study had any systemic involvement or history of anaphylaxis. The result was consistent with the study of Godse et al. that no Indian patient presented with exercise-induced anaphylaxis [6]. To compare with temperate countries, 3 Korean patients (3.3%) with chest tightness were reported by Kim et al. [9]. Respectively, CholU-induced anaphylaxis might be lesser noticed in tropical countries than in temperate countries.

Before 2016, the guideline treatment of physical urticaria was not established. Grattan et al. proposed that patients who failed with single dosage nsAH_1_ could try at least 2 nsAH_1_ together to control chronic urticaria [25]. Alsamarai et al. proposed that the combination of AH_1_ and AH_2_ was an effective treatment of CholU with complete control symptoms and a low relapsing rate [26]. Almost all patients in this study (15 patients) were treated by the combination of AH_1_ (mostly nsAH_1_) and other medications including other nsAH_1_, sAH_1_, AH_2_, and propranolol to control their disease symptom. At present, the current EAACI/GA^2^LEN/EDF/UNEV consensus recommendations for CholU management suggest nsAH_1_ as the first choice of treatment and trigger factor avoidance [1]. If standard dosing is insufficient to control symptoms, updosing of nsAH1 up to 4-fold is recommended. In recalcitrant patients, omalizumab is recommended as the second-line treatment to add with AH_1_ [1, 27]. Currently, novel biologics are being investigated as therapeutic options for CholU [28]. Ligelizumab, a humanized IgG_1_ monoclonal antibody, attaches to the C*ε*3 domain of IgE to inhibit interaction with the FC*ε*RI on the surface of mast cells and basophils that is similar to omalizumab [29]. Quilizumab, a humanized IgG_1_ that binds the M1 prime segment of membrane-expressed IgE, has been demonstrated to decrease IgE levels in human subjects [30]. At present, both of them have been investigated in randomized controlled studies evaluating patients with refractory chronic inducible urticaria including CholU.

The Kaplan-Meier survival analysis demonstrated that the remission rate within 1 year, 5 years, and 13 years was 12.5%, 35.5%, and 67.9% of our CholU patients, respectively. After onset, the median duration before half of the patients were in remission was 12.2 years. Limitations of our study were a limited number of patients with CholU and a retrospective study in which some data was missing. Further studies are needed to investigate rates of remission of CholU in other populations.

## 5. Conclusion

The prevalence of CholU is different in each geographic region, and the prevalence of CholU was found to be low in a tropical country with the median duration of CholU 4.3 years. The prevalence of atopy and anaphylaxis in patients with CholU in tropical countries is lower than that in temperate countries; however, other factors are indistinguishable between the tropical and temperate zones.

## Figures and Tables

**Table 1:** Characteristics and course of cholinergic urticaria in our 16 patients.

Characteristics	Number (%)
Sex	
Male	9 (56.3%)
Female	7 (43.7%)
Mean age of onset (yr)	28.0 ± 11.7 (15, 56)
Mean duration of cholinergic urticaria (yr)	4.9
Associated other urticaria	
Chronic spontaneous urticaria	4 (25.0%)
Dermographism	3 (18.8%)
Cold urticaria	2 (12.5%)
Cold urticaria and dermographism	1 (6.3%)
Angioedema	1 (6.3%)
History of anaphylaxis	0
Personal history of atopy	3 (18.8%)
Allergic rhinitis	2 (12.5%)
Asthma	1 (6.3%)
Allergic conjunctivitis	0
Atopic dermatitis	0
Treatment^#^	
sAH_1_	
Hydroxyzine (2)^∗^	4 (25%)
nsAH_1_	
Cetirizine (1-2)^∗^	7 (43.8%)
Loratadine (1-2)^∗^	6 (37.5%)
Fexofenadine (1-2)^∗^	12 (75%)
Levocetirizine (1)^∗^	1 (6.3%)
Desloratadine (1-2)^∗^	2 (12.5%)
Bilastine (2)^∗^	2 (12.5%)
AH_2_	
Ranitidine (2)^∗^	4 (25%)
Remission (at the time of study)	
Yes	6 (37.5%)
No	10 (62.5%)
Mean disease duration (in 6 cases with remission, yr)	4.3 ± 4.5 (0.3, 13.0)

The data were shown as mean ± standard deviation (minimum, maximum) or number (%). Abbreviations: AH_2_: H_2_ receptor antagonist; CSU: chronic spontaneous urticaria; nsAH_1_: nonsedating H_1_ antihistamine; sAH_1_: sedating H_1_ antihistamine; yr: year. ^#^One patient might have one or more medications. ^∗^The number of tablets per day.

**Table 2:** Comparison of clinical features and course of cholinergic urticaria demonstrated by our study and previous studies^∗^.

Author (yr) (study design)	City (country)	Latitude	Climate zone	Average temperature in summer (°C)	Prevalence of CholU among all chronic urticaria cases (%)	No. (case)	Male (%)	Mean age at symptom onset (yr)	Atopy (%)	History of anaphylaxis (%)	Concomitant form of other urticaria	Mean disease duration^#^ (yr)
Tropical zone												
This study (2019) (retrospective study)	Bangkok (Thailand)	13.7°N	Tropical	35.4	0.7%	16	56.3	28	18.8	0	CSU 25% Dermographism 18.8% Cold urticaria 12.5% Cold urticaria and dermographism 6.3%	4.3
Sánchez et al.^19^ (2017) (prospective study)	Medellín	6.3°N	Tropical	28.5	2%	5	N/A	N/A	N/A	N/A	Dermographism 60%	4
	Bogotá (Colombia)	4.7°N	Subtropical	20.2								
Godse et al.^6^ (2013) (cross-sectional study by questionnaire survey)	Western India	23.9°N	Tropical	40	4.16%	25	56	N/A	N/A	0	CSU 8%	N/A

Temperature zone												
Seo1 et al.^8^ (2019) (retrospective study)	Chuncheon (Korea)	37.8°N	Temperate	25.7	0.023%	11,429	42	N/A	N/A	N/A	N/A	N/A
Kim et al.^9^ (2014) (retrospective study)	Seoul (Korea)	37.5°N	Temperate	25.7	N/A	92	100	27.8	16.3	3	Dermographism 6.5% Cold urticaria 3.3% Food-induced urticaria 2.2%	4
Zuberbier et al.^7^ (1994) (cross-sectional study by questionnaire survey)	Berlin (Germany)	52.5°N	Temperate	24	11.2%	55	43.6	N/A	45.5%	0	N/A	N/A
Asady et al.^2^ (2017) (retrospective study)	Berlin (Germany)	52.5°N	Temperate	24	N/A	200	42	28.1	102	N/A	Another form of chronic urticaria 37.5% (mostly CSU 24.5%)	4.6

Abbreviations: CholU: cholinergic urticaria; CSU: chronic spontaneous urticaria; N/A: not available. ^∗^Excluding studies that did not provide adequate information. ^#^Mean disease duration ranges from onset of symptom to disease remission (in case of remission).

## Data Availability

All research data and supporting information are included within the article.
